# Maternal Thymus Adaptations and Hormone Regulation During Pregnancy

**DOI:** 10.3390/cells14191534

**Published:** 2025-09-30

**Authors:** Ling Yang, Xinxin Wang, Leying Zhang

**Affiliations:** School of Life Sciences and Food Engineering, Hebei University of Engineering, Handan 056038, China; W875016473@163.com (X.W.); zhangly056000@126.com (L.Z.)

**Keywords:** hormones, immunology, pregnancy, thymus

## Abstract

The thymus is necessary for the development of T lymphocytes and central tolerance, and adaptations in the maternal thymus are required during pregnancy. In the present paper, maternal thymic cellular anatomy, T-cell development in the thymus, and related progress are reviewed. In addition, the recent progress in maternal thymic adaptations during pregnancy is discussed, including adaptations in thymic cellular anatomy, T-cell development, and immune-related cytokines. Finally, the latest information about hormones that regulate thymic immunology during pregnancy is summarized. In summary, there are many factors, including a lot of hormones, which are involved in maternal thymic immunological adaptations during pregnancy, and may be used to prevent pregnancy-related thymic diseases and preterm birth.

## 1. Introduction

In humans, there are 5 to 18% of pregnancies associated with preterm birth, and the breakdown of pregnancy immune tolerance at the maternal–fetal interface is a leading cause of preterm birth [1]. The thymus plays a central role in the lymphoid system, which is essential for lymphocyte production and normal immune functions [2]. The development of T lymphocytes in the thymus is associated with immune responses and central tolerance in humans [3]. The thymic microenvironment mediates the maturation and selection of T-cells, which is related to thymic epithelial cells (TECs), dendritic cells (DCs), and mesenchymal cells [4]. T-cell development is dependent on the TECs within the cortical and medullary areas, and non-epithelial stromal cells indirectly regulate TEC development and/or function to influence T-cell development [5].

There are adaptations in the maternal immune system, including the thymus, during successful pregnancy, and the osteoclast differentiation receptor and female sex hormones are involved in regulating the development of thymic regulatory T-cells during pregnancy [6]. Pregnancy increases the expression of progesterone receptor (PGR) in the cortical TECs, which is necessary for thymic involution during murine pregnancy [7]. The thymus undergoes significant involution, a profound loss of early thymic progenitors, as well as decreases in thymocyte proliferation and thymic emigrants, which are associated with the elevating levels of hormones, such as estrogen, during normal pregnancy [8]. Progesterone contributes to the severe reduction in thymus size and thymocyte output, which is involved in maternal–fetal tolerance during pregnancy [9]. It has been reported that pregnancy changes the expression of the complement system, nod-like receptors, nuclear factor kappa B subunits, ikappaB protein, toll-like receptors, interferon-stimulated genes, and prostaglandin synthases in the maternal thymus [10,11,12,13,14,15]. The changes are essential for the maternal immune regulation and successful pregnancy in an animal model.

The maternal thymic immune adaptations during pregnancy are necessary for the mother to avoid detrimental immune responses against the allogeneic fetus, and prevent her thymic diseases related to pregnancy. However, at present, there is no systemic review that focuses on the maternal thymus adaptations during pregnancy. In the present review, thymic cellular anatomy and T-cell development in the thymus are first reviewed. Furthermore, the research progresses and the maternal thymic adaptations during pregnancy are summarized. Finally, the hormones that regulate thymic immunology during pregnancy are highlighted, which may be used to enhance pregnancy rate in humans and animals.

This review was conducted following systematic review processes and standards described in the updated guideline for Preferred Reporting Items for Systematic Reviews (PRISMA) using the electronic bibliographic databases (Pubmed (https://pubmed.ncbi.nlm.nih.gov/ (accessed on 29 August 2025)) and Web of Science (https://www.webofscience.com/wos/ (accessed on 29 August 2025)). Outcomes of interest were impacts on thymus, thymic cellular anatomy, pregnancy, and hormones. After the removal of duplicates and screening of literatures in fetus, infancy, male, and aging, as well as not in mammals, a total of 106 studies were included in this review.

## 2. Adult Thymic Cellular Anatomy

Thymic mesenchymal septae divide the thymus into two lobes that consist of cortical and medullary areas, and specialized stromal cells make up microenvironments, which influence the maturation program of immature T-cell precursors [16]. The thymus comprises subcapsule, cortex, corticomedullary junction (CMJ) and medulla, DCs, vasculature, and mesenchymal cells, and TECs include cortical TECs (cTECs) and medullary TECs (mTECs) [17]. The lymphoid precursors from the bone marrow enter the thymus via blood vessels in CMJ which is related to self-tolerance [18]. Thymocyte development depends on interactions with thymic microenvironments, which include cTECs, mTECs, endothelial cells, mesenchymal/fibroblast cells, DCs, and B cells in the cortex and medulla. In addition, the thymus plays crucial roles in thymocytes development and differentiation, and generates mature T-cells able to respond to foreign antigens but maintains tolerance to self-antigens [19].

## 3. T-Cell Development in the Thymus

**Figure 1:**
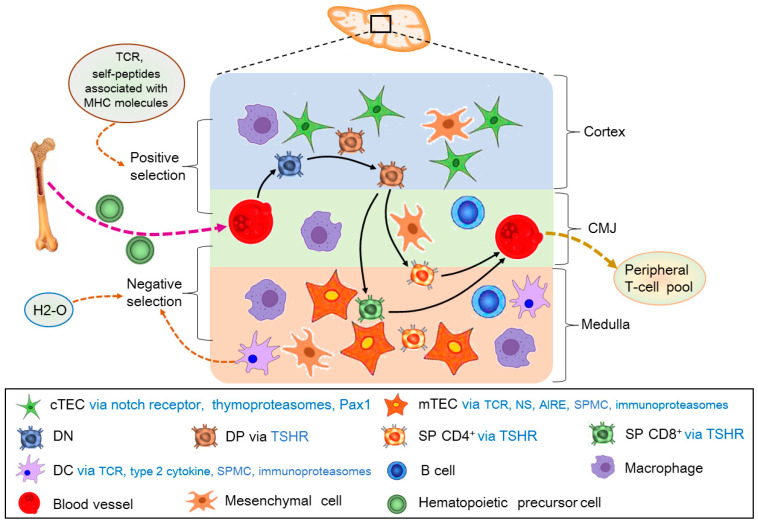
T-cell development in the thymus. First of all, hematopoietic precursor cells from the bone marrow enter the thymus at corticomedullary junction (CMJ) and migrate to the cortex to constitute the CD4^−^ CD8^−^ double negative (DN) thymocytes via interacting with cortical thymic epithelial cells (cTECs). Secondly, DN thymocytes become CD4^+^CD8^+^ double positive (DP) in the cortex under the interaction between cortical microenvironments. The positive selection is involved in cTECs via notch receptor, thymoproteasomes, and transcriptional regulatory protein (Pax1), as well as T-cell receptor (TCR) and self-peptides associated with MHC molecules. Finally, the DP thymocytes mature into single-positive (SP) CD4^+^ or CD8^+^ T-cells in the medulla under the interaction between medullary microenvironments. The negative selection is related to medulla thymic epithelial cells (mTECs) via TCRs, self-peptide MHC (SPMC), autoimmune regulator (AIRE) and immunoproteasomes, DC via TCR, type 2 cytokine, SPMC and immunoproteasomes, and MHC class II peptide-editing molecular chaperon (H2-O). SP CD4^+^ or CD8^+^ T-cells emigrate from the thymus through CMJ to establish the peripheral T-cell pool. In addition, DP, SP CD4^+^, and CD8^+^ T-cells are under the influence of thyroid-stimulating hormone (TSH) via its receptor (TSHR).

T and B cells share a common lymphoid progenitor, and B cells develop in the confines of the bone marrow. However, T-cell development occurs in the thymus that provides specialized stromal or epithelial cells, as well as unique signals, which are required for proper thymocyte differentiation [20]. T-cells undergo a series of differentiation steps defined based on the cell-surface expression of CD4 and CD8. At the first step, hematopoietic precursor cells from the bone marrow enter the thymus at CMJ and migrate to the outer cortex to constitute the CD4^−^ CD8^−^ double negative (DN) thymocytes via interacting between notch receptor-expressing cortical epithelial cells. Secondly, DN thymocytes become CD4^+^CD8^+^ double positive (DP) in the cortex under the interaction between cortical microenvironments. Finally, the DP thymocytes mature into single-positive (SP) CD4^+^ or CD8^+^ T-cells in the medulla under the interaction between medullary microenvironments [21]. SP CD4^+^ or CD8^+^ T-cells emigrate from the thymus to establish the peripheral T-cell pool (Figure 1).

The cTECs are involved in the positive selection of thymocytes, whereas the mTECs support negative selection to induce self-tolerance [22]. Type 2 cytokines activate Sirpα^+^ DCs to mediate thymocyte selection in the thymus, which enforces central tolerance [23]. The autoimmune regulator expressed by mTECs plays a critical role in self-tolerance, which is through deleting autoreactive T-cells and promoting thymic regulatory T (T_reg_)-cell development in the thymus [24]. Thymoproteasomes expressed by cTECs are essential for optimal positive selection of CD8^+^ T-cells, whereas immunoproteasomes expressed by mTECs contribute to the establishment of self-tolerance in the T-cells. In addition, immunoproteasomes expressed by DCs and developing thymocytes are associated with T-cell development in the thymus [25].

As a major histocompatibility complex (MHC) class II peptide-editing molecular chaperon, H2-O has effects on the selection of thymic Tregs. H2-O deficiency in the thymic medulla promotes regulatory T-cell differentiation and increases basal auto-stimulation of CD4 T-cells [26]. The affinity between T-cell receptors and self-peptides associated with MHC molecules mediates positive T-cell selection in the thymic cortex [27]. The fine-tuning of key transcriptional regulators downstream of TCR signaling can control innate-like γδ T-cell effector commitment in the thymus [28]. TCRs expressed by T-cell precursors interact with self-peptide MHC complexes in the thymic mTECs and DCs directly and indirectly, which is implicated in the T-cell selection process [29]. The mature B cells act as specialized antigen-presenting cells in the thymic medulla, which mediate the negative selection of self-reactive T-cells in the thymus [30]. Self-reactive SP cells are deleted by ‘negative selection’, mediated by thymic DCs and mTECs [22]. As a transcriptional regulatory protein, Pax1 expressed in adult cTECs is necessary for establishing the thymus microenvironment, which is required for normal T-cell maturation and maintaining the total size of the thymus [31]. Therefore, there are lots of factors that are involved in T-cell development in the thymus.

## 4. Maternal Thymic Adaptations During Pregnancy

During pregnancy, the thymic cortex shrinks, and the medulla enlarges and rearranges, which creates a microenvironment containing increased numbers of mature thymocytes, and contributes to the immune suppression of the mother to paternal and fetal antigens [32]. The thymus shrinks in size and the cortex at the ultrastructural level, and the epithelial cells of the subcapsular cortex become wrinkled and more phagocytosis occurs. Thymus and body weight during pregnancy and the number of progeny are affected by strain difference in the rat [33]. In addition, the mTECs increase in mitosis, and DCs in the medulla become more conspicuous and phagocytic during pregnancy in mice [34]. The number of T helper cells and cytotoxic T-cells is lower in the first and third trimesters of pregnancy, but the number of suppressor T-cells is higher in the first trimester of pregnancy compared to pre-pregnancy in humans [35]. Thymus-derived regulatory T-cells play an important immunosuppressive role during pregnancy, which is essential for maternal–fetal tolerance [36].

**Figure 2:**
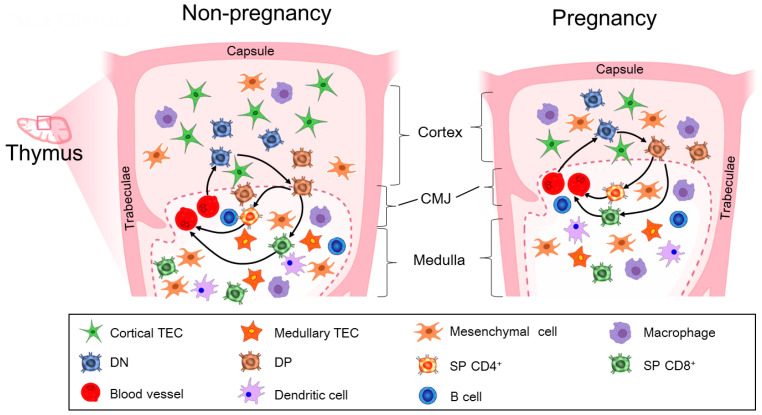
Pregnancy induces changes in the thymic cortex and size, the medulla, and cell populations. During pregnancy, the thymic cortex and size shrink, but the medulla enlarges and rearranges, and populations of cTECs, mTECs, and mesenchymal cells decease. In addition, populations of the CD4^−^ CD8^−^ double negative (DN), CD4^+^CD8^+^ double positive (DP), and single-positive (SP) CD4^+^ or CD8^+^ T-cells also decrease. These changes are involved in the modulation of T-cell development in the thymus.

Maternal thymus cell populations and mitogenic responsiveness are significantly changed during pregnancy [37]. Pregnancy results in profound loss of early thymic progenitors and suppressed proliferation of developing thymocytes in the mammalian thymus [8]. Gonadotropin-releasing hormone (GnRH) and patterns of prohibitin are implicated in pregnancy-induced thymic involution, which is important for the maturation of T lymphocytes during pregnancy in rats [38]. Pregnancy-induced thymic involution reduces all major thymic lymphoid cell populations, including the early T-lymphoid progenitors, thymic regulatory T-cells, and all major nonlymphoid cell populations. In addition, pregnancy also decreases the expression of chemokines in the thymic nonlymphoid cells and can be downregulated by short-term treatment with progesterone but not estrogen [9]. There is an upregulation of PGR in the cTECs during pregnancy, which is related to thymocyte maturation and thymic involution in mice [7]. Thymic adaptations to the semi-allogeneic fetus lead to acute thymic involution during pregnancy, which is mainly induced by progesterone, and associated with the shrinkage of thymus volume [39]. Pregnancy induces shrinking in the thymic cortex and size, and enlarges and rearranges in the medulla, deceasing in populations of cTECs, mTECs, and mesenchymal cells (Figure 2).

Maternal T_Regs_ derived from the thymus suppress reactive effector T-cells, which contribute to the potential role of maternal–fetal tolerance during successful pregnancy [40]. Pregnancy reduces the output of recent thymic emigrant-regulatory T-cells (RTE-Treg), decreases the ratio of RTE-Treg/mature naïve Tregs, which contributes to maternal immune tolerance to the semi-allogeneic fetus and the maintenance of pregnancy in humans. In addition, pregnancy changes the expression of CD4 protein [41], and modulates the expression of helper T cytokines, including tumor necrosis factor beta, interferon-gamma, interleukin-2 (IL-2), IL-4, IL-5, IL-6, and IL-10 in the ovine maternal thymus [42].

Pregnancy causes a severe decrease in the number of all thymocyte subsets and qualitative changes in cTECs, which is triggered by progesterone. However, *Klf4* can protect cTEC’s integrity, and mitigate thymic involution during late pregnancy [43]. The production of DN T-cells is mediated by estrogen receptor-α (ERα) located in thymic mast cells, and the numbers of DN T-cells increase dramatically in pregnant females. In addition, the cytokines produced by gamma/delta DN T-cells are necessary for the maintenance of pregnancy [44]. Apart from regulatory T-cells, DN T regulatory cells also participate in the immune regulation and tolerance of the female reproductive system, which is essential for ovulation, implantation, and pregnancy maintenance [45]. The thymus is involved in the development of CD4^+^Foxp3^+^ TReg cells that have capital effects on modulating embryo implantation and fetal growth by progesterone in mice [46]. In addition, the autoimmune regulator is expressed in mTECs, which is essential for thymic selection and maintaining maternal–fetal tolerance in humans and mice [47].

**Figure 3:**
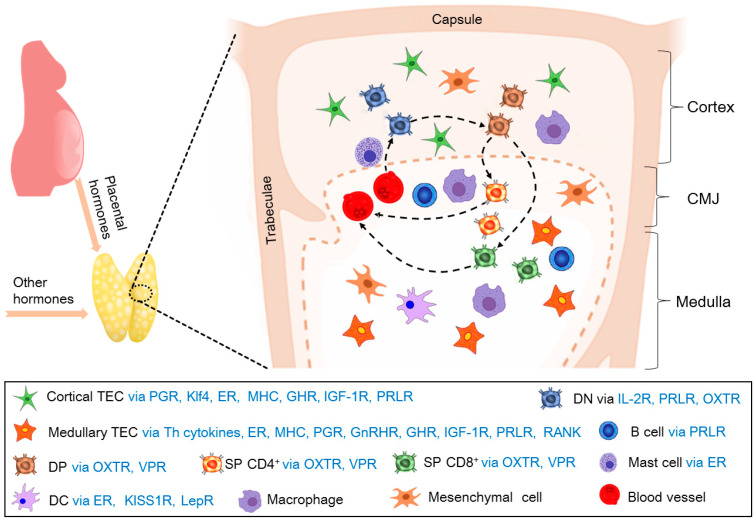
T-cell development is regulated in the maternal thymus during pregnancy. During pregnancy, T-cell development under the thymic microenvironments is regulated by cTECs via PGR, Klf4, ER, MHC, GHR, IGF-1R, and PRLR, and mTECs via Th cytokines, ER, MHC, PGR, GnRHR, GHR, IGF-1R, PRLR, and RANK. In addition, the microenvironments are modulated by B cells via PRLR, mast cells via ER, and DCs via ER, KISS1R, and LepR. Furthermore, T-cell development is regulated in autocrine or paracrine manners through DN T-cells via IL-2R, PRLR, OXTR, DP T-cells via OXTR, VPR, and SP CD4^+^ and SP CD8^+^ T-cells via OXTR and VPR.

During pregnancy, T-cell development under the thymic microenvironments is regulated by cTECs, mTECs, B cells, and mast cells, which are via many cytokines to regulate maternal thymic adaptations. In addition, the T-cell regulates its development in an autocrine or paracrine manner (Figure 3). However, more studies on the cytokines related to maternal thymic adaptations are needed.

## 5. Hormones That Regulate Thymic Immunology During Pregnancy

**Figure 4:**
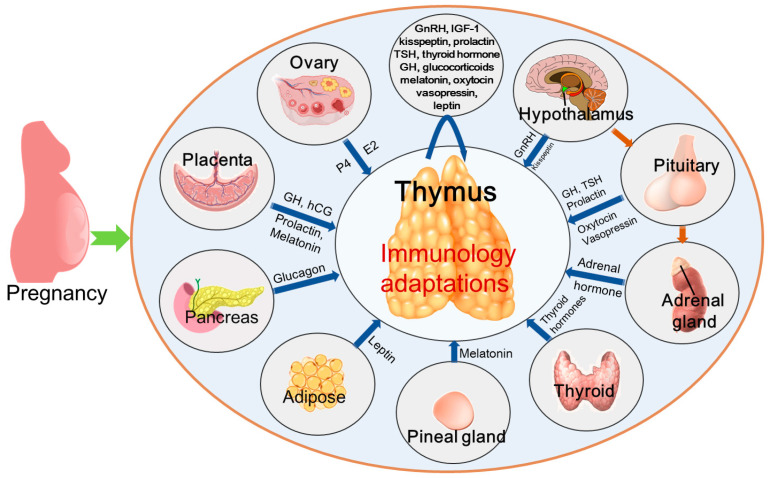
Hormones regulate maternal thymic immunological adaptations during pregnancy. During pregnancy, placental hormones (including human chorionic gonadotropin (hCG), growth hormone (GH), prolactin, and melatonin) exert effects on the maternal thymus. In addition, ovaries (progesterone (P4) and estrogen (E2)), hypothalamus (gonadotropin-releasing hormone (GnRH) and kisspeptin), and the pituitary gland (GH, thyroid-stimulating hormone (TSH), prolactin, oxytocin, and vasopressin), participate in regulating maternal thymic immunological adaptations during pregnancy. Furthermore, pineal gland (melatonin), adrenal glands (adrenal hormone), pancreas (insulin and glucagon), thyroid (thyroid hormones) and white adipose tissue (leptin) are also involved in maternal thymic immunological adaptations. Moreover, GnRH, insulin-like growth factor 1 (IGF-1), kisspeptin, prolactin, TSH, thyroid hormone, GH, glucocorticoids, melatonin, oxytocin, vasopressine, leptin, and insulin are produced by the thymus, which regulate thymic immunological adaptations in an autocrine or paracrine manner.

Hormones act on thymic microenvironment cells and thymocytes in both endocrine and paracrine/autocrine pathways, which modulate the proliferation and survival of thymic microenvironment cells, selection of the T-cell repertoire, as well as the migration and export of developing T-cells. The hormones include growth hormone (GH)/insulin-like growth factor 1 (IGF-1), prolactin (PRL), leptin, thyroid hormone, sex hormones, and glucocorticoids [48]. The hormones that regulate thymic immunology during pregnancy include human chorionic gonadotropin (HCG), estrogen, progesterone, GnRH, GH/IGF-1, kisspeptin, PRL, thyroid-stimulating hormone (TSH), thyroid hormone, glucocorticoids, melatonin, oxytocin, vasopressin, leptin, and insulin (Figure 4). However, it is also possible that other hormones are involved in modulating thymic immunology during pregnancy.

### 5.1. Human Chorionic Gonadotropin

HCG has multiple endocrine, paracrine, and autocrine actions on a variety of gestational cells and tissues, including the immune system, to promote and maintain pregnancy in humans [49]. HCG administration inhibits thymic T-cell development and peripheral T-cell populations, which has negative effects on immune function in mice [50]. Chorionic gonadotrophin affects the thymic secretory function in pubertal female mice [51], and HCG enhances the effect of hormones on the in vitro differentiation of thymocytes in the presence of TECs in humans [52]. However, HCG does not affect the phenotype of the human thymocytes but influences the production of autocrine growth factors by these cells, which regulates antigen-independent differentiation of T-lymphocytes during pregnancy [53]. HCG is detectable from 10 days after fertilization, increased to a peak in weeks 9 to 10 of pregnancy, and subsequently declined. HCG can increase the frequency of Treg cells and restrict pregnancy-harmful proinflammatory Th17 responses in mice [54]. Therefore, HCG has effects on thymus functions, and further research is needed on the molecular mechanism that HCG regulates for thymic function during pregnancy.

### 5.2. Estrogen

Estrogens can modulate atrophy and phenotypic alterations of the thymus, and T-cell development via ERα [55]. Estrogen treatment can inhibit the production of thymic factors and change T-cell subpopulations via its receptors in the thymus to modulate thymic immune function [56]. In addition, estrogen regulates the development and differentiation of T-cells and the immune functions of the TECs through ERs, which contributes to autoimmunity [57]. Estriol modulates the processes of myeloid DC maturation in the thymus during the first half of pregnancy and also is an element of steroid-induced involution of the thymus during this period [58]. In addition, estrogen has effects on thymocytes and thymic stromal cells (mainly TECs), which are involved in the modulation of T-cell development and repertoire selection for central tolerance [59].

Estrogen treatment reduces thymic size and cellularity, as well as defined T-cell subsets of CD4 and CD8 in the mouse thymus [60]. The high serum level of estradiol inhibits the percentage of CD4^+^CD8^+^ DP T-cells in the mouse thymus during the proestrus phase, but CD4^+^CD8^−^ or CD4^−^CD8^+^ SP T-cells are significantly increased in the proestrus phase via ERα [61]. Basal estrogens promote normal thymus growth but inhibit cortical double negative development via ERα, and reduce expression of MHC in the TECs [62]. The elevated estrogen levels during pregnancy reduce thymocyte proliferation, which contributes to thymic involution and loss of thymocyte cellularity during pregnancy [8]. In addition, early pregnancy regulates the expression of ERα and ERβ in the maternal thymus, which is involved in the regulation of maternal immune function during early pregnancy in ewes [63]. Thus, estrogen is related to the establishment of immune tolerance during normal pregnancy, and expression of ERα and ERβ in the maternal thymus during mid and late pregnancy needs to be investigated further.

### 5.3. Progesterone

There are specific intracellular PGRs that interact with progesterone to perform pleiotropic reproductive activities of progesterone in the female thymus [64]. Progesterone has effects on thymulin-secreting TECs, which modulates proliferation, secretion, and the degree of interactions between TECs and thymocytes through both cytoplasmic and membrane-bound PGRs [65]. Pregnancy induces the expression of PGR in thymic stromal cells (mainly TECs), which is specifically essential for thymic involution and block of T-cell development [66]. Progesterone promotes the development of thymic Treg cells through differentiation receptor RANK in mTECs during pregnancy, and depletion of RANK in the mouse thymic epithelium results in fetal loss and maternal glucose intolerance [6]. It has been reported that there is an upregulation of the 60-kDa PGR isoform and the 62-kDa progesterone-induced blocking factor variant in the maternal thymus during early pregnancy in ewes [67], and the upregulation of PGR in maternal cTECs during pregnancy is required for thymic involution and successful pregnancy in mice [7]. Therefore, upregulation of PGR in the maternal thymus is essential for normal pregnancy, but the molecular mechanism that progesterone regulates, thymic involution, needs to be studied further.

### 5.4. GnRH

GnRH is mainly secreted by the hypothalamus and also synthesized by the thymus, which regulates the development and function of thymic T lymphocytes [68]. There is a thymus–hypothalamus–pituitary–gonadal axis through which the thymus gland secretes factors to regulate the release of GnRH, the production of luteinizing hormone, and the secretion of gonadal steroids [69]. Both GnRH and GnRH receptors (GnRHRs) are mainly expressed in the thymic medulla, which exert direct actions on the immune modulation in the immune cells [38,70]. GnRHR agonist leuprolide treatment induces immunosuppression, which is related to the levels of thymus nitric oxide and changes in immunological parameters [71]. GnRHR agonist leuprolide treatment also enhances thymus weight and increases secretion of thymosin alpha 1 in the thymus [72]. Furthermore, GnRH agonist infusions attenuate pregnancy-induced thymic involution, and increase thymic weight in pregnant rats. On the other hand, the maternal thymus expresses GnRH and GnRHR, and early pregnancy can stimulate the expression of GnRH, but inhibit the expression of GnRHR in the maternal thymus in sheep [73]. Taken together, these findings suggest that GnRH and GnRHR are involved in modulating maternal thymus function during pregnancy. Thus, further research may focus on the molecular mechanism that GnRH regulates, maternal thymus function, during pregnancy.

### 5.5. Growth Hormone/Insulin-Like Growth Factor 1

GH acts directly on target cells or indirectly by stimulating the production of IGF-1 to promote body growth and metabolism. GH and IGF-1 are expressed in the thymus to regulate the development and function of immune cells and thymus involution [74]. GH can improve thymocyte proliferation and migration, and also affect the secretion of cytokines and thymic hormones in humans and mice [75]. GH is involved in the regulation of the thymic microenvironment [48], which is mediated by IGF-1 in the thymus [75]. GH and IGF-1 secreted by human thymocytes enhance the proliferation of thymocytes and TECs to influence immune function [76]. The IGF-1 receptor is expressed in human thymic cells and murine TECs and thymocytes, which are involved in intrathymic T-cell differentiation and migration [77]. Furthermore, GH enhances total thymocyte numbers and the secretion of thymulin from TECs mediated by IGF-1, which are via GH and IGF-1 receptors on thymocytes and TECs [78]. Exogenous IGF-1 improves thymopoiesis through TECs expansion in mice [79]. The IGF-1 receptor expressed in the thymus is involved in thymic rejuvenation and involution in pigs [80]. The placental GH increases, and gradually replaces pituitary GH, and maternal IGF-1 levels also upregulate during pregnancy [81]. On the other hand, the changes in the expression of GH and GH receptors in the maternal thymus are implicated in the adaptations of the maternal thymus in ewes [82]. Thus, GH and IGF-1 participate in modulating maternal thymus function during pregnancy. However, the molecular mechanism that GH and IGF-1 modulate for thymic adaptation during pregnancy needs to be explored further.

### 5.6. Kisspeptin

Kisspeptin is secreted by the hypothalamus, and is also found in extra-hypothalamic areas, which is a critical regulatory factor of GnRH release [83]. The *KISS1* gene encodes kisspeptins that bind to kisspeptin receptors, including GPR54 [84], and *GPR54* mRNA, which are expressed in the thymus [85]. *GPR54* deficiency results in thymus enlargement, an increase in thymocytes, and altered thymic micro-architecture, which is involved in T-cell development and self-tolerant immunity in mice [86]. Kisspeptin has effects on the processes of differentiation of thymic myeloid DCs during the second–third trimesters [58]. There is a dramatic increase in circulating levels of kisspeptin during a healthy pregnancy, which plays a fundamental role in the regulation of GnRH secretion and placentation [87]. Thus, the upregulation of kisspeptin levels is implicated in the T-cell development of the maternal thymus during pregnancy.

### 5.7. Prolactin

Lactotroph cells in the anterior pituitary gland synthesize PRL, and the principal role of PRL is to regulate lactation via the PRL receptor (PRLR) [88]. PRL has beneficial effects on the survival and differentiation of T-cell progenitors [89], and administration of PRL antiserum modulates the developmental pattern of T-lymphocytes in the thymus [90]. In addition, PRL can stimulate the secretion of thymulin by TECs, and maintain thymocyte viability during the DP stage of thymocyte differentiation, which is through PRLR on thymocytes and TECs [79]. Developing thymocytes produce PRL, and PRLR is expressed in T-cells, TECs, and B cells [91], and PRL treatment has effects on the migration of total DP, CD4-positive, and CD8-positive thymocytes [92]. It has been reported that there is an upregulation of PRL and PRLR in the maternal thymus during early pregnancy in ewes, and PRLR protein is located in the epithelial reticular cells, capillaries, and thymic corpuscles [93]. Therefore, the changes in the expression of PRL and PRLR in the maternal thymus are associated with T-cell development and proliferation during early pregnancy. However, the effect of PRL on the maternal thymus during mid and late pregnancy needs to be studied further.

### 5.8. Thyroid-Stimulating Hormone and Thyroid Hormone

TSH can bind and activate the TSH receptor (TSHR) in thymocytes to enhance T-cell development, and lacking functional TSHR expression is associated with lower frequencies of DP and SP thymocytes in mice [94]. It has been reported that β-TSH is expressed in the subcapsular and cortical thymic zones [95], and TSH increases the frequencies of CD4^+^ and CD8^+^ SP thymocytes, and protects thymocytes from apoptosis [96].

Triiodothyronine has a pleiotropic effect on thymus physiology [97], and triodothyronine treatment of cultured TECs increases thymulin synthesis and secretion in mice [98]. On the other hand, the thyrotropin receptor is expressed in the thymus, which enhances thymocyte proliferation in an autocrine/paracrine manner [48]. Free thyroxine levels increase during the first trimester and then decrease after the first trimester. However, TSH concentrations decrease, and then return to normal during the same stages [99]. Therefore, TSH and thyroid hormone, as well as their receptors, are expressed in the maternal thymus, which are related to T-cell development and thymocyte output. However, the effects of TSH and thyroid hormone on the maternal thymus during pregnancy need to be studied.

### 5.9. Glucocorticoids

Glucocorticoids are mainly synthesized in the adrenal glands and have anti-inflammatory and immune-suppressive actions [100]. The thymus also produces glucocorticoids which are critical modulators in the immune system [101]. Endogenous glucocorticoids have pleiotropic effects on different T-cell populations [102], and endogenous glucocorticoids play a positive role in thymocyte selection via glucocorticoid receptors (GRs) [103]. Elevated serum glucocorticoid levels induced by stress responses result in thymocyte apoptosis and thymic involution in humans and mice [104]. Glucocorticoid treatment increases the production of immunosuppressive cytokines by thymic Treg cells and immunosuppressive cytokine expression, which contributes to the immunosuppressive effects of glucocorticoids [105]. Maternal glucocorticoid concentrations critically rise during pregnancy, which promotes immune tolerance via GR-mediated pathways [106]. Taken together, glucocorticoids are associated with maternal immune-suppressive actions, but the function of glucocorticoids in the maternal thymus during pregnancy needs to be investigated further.

### 5.10. Melatonin

The pineal gland is the major site for melatonin production, and melatonin is also synthesized by other organs, including the thymus. Melatonin exerts its effects through melatonin receptors expressed in many mammalian organs [107]. Endogenous thymic melatonin synthesis is regulated by circulating levels of melatonin [108], and melatonin treatment in physiological doses exerts effects on cell proliferation and differentiation of T-cells in the thymus [109]. In addition, melatonin treatment decreases mast cell densities in the thymus, which is involved in immune system regulation and proinflammatory cytokine production [110]. There is a progressive increase both in maternal and placental melatonin levels during normal pregnancy [111]. On the other hand, melatonin receptors are upregulated in the maternal thymus during early pregnancy, which is related to immune regulation of the maternal thymus in an animal model [41]. Thus, the progressive increase in maternal and placental melatonin levels may be involved in immunoregulation of the maternal thymus via melatonin receptors during pregnancy, which needs to be investigated further.

### 5.11. Oxytocin and Vasopressin

Oxytocin and vasopressin released by the neurohypophysis have effects on thymus physiology, and their transcripts are found in the thymus. In addition, oxytocin receptors are detected in all thymocyte subsets, whereas vasopressin receptor is only found in DP and SP CD8 cells [112]. Oxytocin and vasopressin are peptides with high physiological relevance, and play a central role in bodily homeostatic regulation via banding to oxytocin receptors and vasopressin receptors [113]. Oxytocin and vasopressin play a role in regulating the thymus microenvironment [114], and oxytocins, including intrathymic oxytocin, are involved in the central immunological self-tolerance of T-cells [115]. Inhibition of the oxytocin receptor is related to apoptosis of CD8^+^ mature T-cells, while the vasopressin receptor is associated with T-cell differentiation in the thymus [48]. Neurophysin levels of vasopressin and oxytocin elevate, and the release of these hormones also increases during pregnancy [116]. Oxytocins released from the neurohypophysis and peripheral organs (including the thymus) play a key role in the initiation of pregnancy in rodents [117]. However, the function of vasopressin in the maternal thymus during pregnancy needs to be investigated further.

### 5.12. Leptin

Leptin is mainly synthesized in white adipose tissue [118]. The thymus also expresses leptin, which has a protective effect on thymocytes from apoptosis via the leptin receptor in the thymus [48]. The intrathymic role of leptin includes maintaining healthy thymic epithelium and promoting thymopoiesis [119], and leptin can regulate thymic plasmacytoid DC abilities, and influence the thymocyte distribution [120]. There is an increase in the leptin concentration in the maternal circulation during pregnancy, which contributes to adaptations of the energy demands of the growing fetus [121]. An immunosuppressive fraction of boar seminal vesicle fluid lowers the concentration of leptin in blood plasma and adipose tissue, which prevents thymus involution during the first period of pregnancy in mice [122]. In addition, as a proinflammatory adipocytokine, leptin can improve thymopoiesis and regulate T-cell immune responses, which is involved in restoring thymic involution associated with obesity in mice [123]. However, the studies that leptin and its receptors are related to maternal thymus involution during pregnancy in humans and domestic animals need to be investigated further.

### 5.13. Insulin

Insulin can be used for the management of hyperglycemia and also serves as an immunomodulatory hormone [124]. Insulin is expressed in the thymus, which is related to the induction of immune tolerance [125], and susceptibility to type 1 autoimmune diabetes in humans and mice [126]. In addition, thymic insulin levels play a pivotal role in the self-tolerance of insulin-specific T-cells in mice [127]. Insulin expressed by thymic epithelial cells is involved in trimming and removing high-affinity insulin-specific T-cells in mice [128], and the autoimmune regulator controls the expression of insulin in the thymus in humans [129]. There is an increasing demand for glucose, which results in a rise in insulin levels, as well as site-specific central insulin resistance during pregnancy [130]. On the other hand, early pregnancy modulates the expression of insulin receptor β gene and protein in ewes (our unpublicized data). However, the function of insulin in the maternal thymus during mid and late pregnancy needs to be investigated further.

## 6. Conclusions and Future Prospects

Pregnancy induces maternal thymus adaptations that are necessary for successful pregnancy, which results in thymus involution, changes in major thymic lymphoid cell populations, and T-cell development in the thymus. The factors and cells involved in the maternal thymus adaptations during pregnancy are summarized in Table 1. In addition, the hormones that modulate thymic immunology are reviewed, which may be used for avoiding pregnancy-related thymic diseases, and preventing embryo loss and abortion. However, there are differences in early pregnancy recognition signals among different species and placental types. Therefore, in different species, pregnancy-induced maternal thymus adaptation may vary in early pregnancy. Nevertheless, the difference may not be significant in the middle and later stages of pregnancy. Thus, many mysteries still exist in modulating thymic immune functions at different stages of pregnancy, and more studies in these areas are needed to figure out the regulatory mechanisms of maternal thymic immunology adaptations.

## Figures and Tables

**Table 1:** Summary of the factors and cells in the maternal thymus adaptations during pregnancy.

Factors and Cells	Effects on Thymic Function	Effects of Pregnancy	Species	Ref.
Osteoclast differentiation receptor RANK	Development of thymic regulatory T-cells	Fetal loss and gestational diabetes	Mice	[6]
Nuclear progesterone receptor	Regulation of thymus involution	Increases in expression of progesterone receptor	Mice	[7]
Estrogen and thymocyte	Regulation of thymus involution	Elevating levels of hormones	Mice	[8]
Thymic nonlymphoid cells, CCL25, CXCL12, CCL21, and CCL19	Thymic involution	Maternal–fetal tolerance	Mice	[9]
Toll-like receptor	Thymic immune	Maternal immune tolerance	Sheep	[10]
NF-κB subunits	Thymic immune	Maternal central immune tolerance	Sheep	[11]
Nod-like receptor	Thymic immune responses	Maternal immunomodulation	Sheep	[12]
Complement components	Thymic immune	Maternal immune regulation	Sheep	[13]
IkappaB protein	Thymic immune regulation	Maternal immunologic tolerance	Sheep	[14]
Interferon-stimulated genes	Thymic immune	Maternal immunologic tolerance	Sheep	[67]
Prostaglandin synthases	Thymic immune regulation	Maternal immunologic tolerance	Sheep	[15]
Sex steroids	Cortical involution of the thymus	Immune suppression of the mother to paternal and fetal antigens	Humans and mice	[32]
Strain difference	Thymic weight	Number of progeny	Rats	[33]
Epithelial cells of the subcapsular cortex, mTECs	Thymus shrinks in size and the cortex	Maternal tolerance to fetal antigens	Mice	[34]
Estrogen and progesterone	Thymic involution	Maternal immune system maintains tolerance towards the allogeneic fetus	Humans	[35]
Treg cells	Immunosuppressive role of the thymus	Maternal–fetal tolerance	Mice	[36]
Cell populations	Thymic immune	Maternal immune reactivity	Mice	[37]
Gonadotropin-releasing hormone	Thymic involution	Maturation of T lymphocytes during pregnancy	Rats	[38]
Progesterone	Thymic involution	Adaptations to the semi-allogeneic fetus	Humans	[39]
Thymus regulatory T-cells, T-cell receptor, autoimmune regulator, and mTECs	Thymocyte development and differentiation in the thymus	Maternal–fetal tolerance to the fetus	Humans	[40]
CD4, MT1, and MT2	Thymic immune regulation	Immune regulation of the maternal immune system	Sheep	[41]
Helper T cytokines	Thymic immune regulation	Immune tolerance in maternal immune system	Sheep	[42]
Klf4, thymic epithelial cells	Thymic involution	Maintaining cTEC numbers during pregnancy	Mice	[43]
α/β and γ/δ double negative T-cells	Thymocyte loss and thymic involution	Maintenance of pregnancy	Humans and mice	[44]
Double negative T regulatory cells	Thymic development	Implantation failure, and pregnancy loss	Humans and mice	[45]
Progesterone, CD4^+^Foxp3^+^ TReg cells	Thymic involution	Embryo implantation and fetal growth	Mice	[46]
Aire and mTECs	Thymic selection	Maintaining maternal–fetal tolerance	Humans and mice	[47]
Human chorionic gonadotropin	Antigen-independent differentiation of T-lymphocytes	Production of autocrine growth factors during pregnancy	Humans	[53]
Estriol and kisspeptin	Myeloid DC maturation in the thymus	Maintaining systemic tolerance of the mother	Humans	[58]
Estrogen	T-cell development	Immune suppression for a potential pregnancy	Humans and mice	[59]
Estrogen	Inhibit cortical double negative development	Pregnancy-driven involution	Humans and mice	[62]
Estrogen receptor α and β	Thymic immune regulation	Regulation of maternal immune function		[63]
Progesterone	Thymic involution	Normal fertility	Humans, rats, and mice	[64]
Progesterone	Thymic involution and T-cell development	T-cell lymphopoiesis during pregnancy	Mice	[66]
Progesterone receptor and PIBF	Thymic immunoregulatory functions	Maternal immune tolerance	Sheep	[67]
GnRH and GnRHR	Modulation of thymus function	Blockade of lymphocyte development in maternal thymus	Sheep	[73]
Prolactin and PRLR	Thymic innate immune	Pregnancy increases expression of prolactin and PRLR	Sheep	[93]
Glucocorticoid	Thymic immune tolerance	Pregnancy increases expression of glucocorticoid receptors	Humans and mice	[106]
Oxytocins	Thymic immune regulation	Initiation of pregnancy	Mice and rats	[116]
Leptin	Thymus involution	Loss of thymus mass during pregnancy	Mice	[122]

## Data Availability

Not applicable.

## Abbreviations

AIRE	Autoimmune regulator
CG	Chorionic gonadotrophin
cTECs	Cortical thymic epithelial cells
DCs	Dendritic cells
DN	Double negative
DNTregs	Double negative T regulatory cells
DP	Double positive
ERα	Estrogen receptor-α
GH	Growth hormone
GnRH	Gonadotropin-releasing hormone
GnRHR	Gonadotropin-releasing hormone receptor
GRs	Glucocorticoid receptors
HCG	Human chorionic gonadotropin
IGF-1	Insulin-like growth factor 1
IL-2	Interleukin-2
MHC	Major histocompatibility complex
mTECs	Medullary thymic epithelial cells
NES	Non-epithelial stromal cells
PGR	Progesterone receptor
PRL	Prolactin
RTE-Treg	Recent thymic emigrant-regulatory T-cell
SP	Single-positive
TCRs	T-cell receptors
TECs	Thymic epithelial cells
Th	Helper T
Treg	Regulatory T
TSH	Thyroid-stimulating hormone
TSHR	Thyroid-stimulating hormone receptor
tT_Regs_	T_Regs_ derived from the thymus
